# Prevalence of extended-spectrum-β-lactamase-producing *Enterobacteriaceae*: first systematic meta-analysis report from Pakistan

**DOI:** 10.1186/s13756-018-0309-1

**Published:** 2018-02-20

**Authors:** Samyyia Abrar, Shahida Hussain, Rehan Ahmad Khan, Noor Ul Ain, Hayat Haider, Saba Riaz

**Affiliations:** 1 Department of Microbiology and Molecular Genetics, University of the Punjab, Lahore, Pakistan; 2 Citilab and Research center, Lahore, Pakistan; 3 College of Statistical and Actuarial Sciences, University of the Punjab, Lahore, Pakistan

**Keywords:** MDR, ESBLs, *Enterobacteriaceae*, Meta-analysis, Pakistan

## Abstract

**Background:**

South-Asia is known as a hub for multidrug-resistant (MDR) bacteria. Unfortunately, proper surveillance and documentation of MDR pathogens is lacking in Pakistan. The alarming increase in the prevalence of extended-spectrum β-lactamase (ESBL)-producing *Enterobacteriaceae* is a serious problem. From this perspective, we analysed published data regarding ESBL-producing *Enterobacteriaceae* in different regions of Pakistan.

**Methods:**

A meta-analysis was performed to determine the prevalence of ESBL-producing *Enterobacteriaceae* in Pakistan. A Web-based search was conducted in electronic databases, including PubMed, Scopus and PakMedi Net (for non-indexed Pakistani journals). Articles published (in either indexed or non-indexed journals) between January 2002 and July 2016 were included in the study. Relevant data were extracted, and statistical analysis was performed using the *Metaprop* command of STATA version 14.1.

**Results:**

A total of 68 studies were identified from the electronic data base search, and 55 of these studies met our inclusion criteria. Pakistan’s overall pooled proportion of ESBL-producers was 0.40 (95% CI: 0.34–0.47). The overall heterogeneity was significant (I2 = 99.75%, *p* < 0.001), and significant ES = 0 (Z = 18.41, *p* < 0.001) was found. OXA, SHV, TEM and CTX-M were the most commonly found gene variants for ESBLs in these studies.

**Conclusion:**

The prevalence of ESBL-producing *Enterobacteriaceae* is high in Pakistan. Little is known about the annual frequency of ESBLs and their prevalence in different provinces of Pakistan. No data are available regarding ESBL frequency in Baluchistan. This underscores an urgent demand for regular surveillance to address this antimicrobial resistance problem. Surveillance to better understand the annual ESBL burden is crucial to improve national and regional guidelines.

## Background

Antimicrobial resistance has been declared a global threat to public health, as a massive increase in this problem has been observed in different parts of the world [1]. Although the magnitude of the antimicrobial resistance problem differs by country and geographical region, South-Asia is considered to be a major region for multidrug-resistant (MDR) bacteria [2]. The reported frequency of MDRs is increasing, putting strain on the public health organizations that are attempting to control this issue in many countries [3].

The alarming increase in the prevalence of extended-spectrum β-lactamase (ESBL)-producing *Enterobacteriaceae* has serious consequences for treatment outcomes [4]. *Escherichia coli* and *Klebsiella* spp. are important pathogens isolated from community-acquired and nosocomial-acquired infections, and have been studied extensively [5–22]. The ESBL enzymes produced by these bacteria make them resistant to the first-choice antibiotic therapies that are commonly used. ESBL-positive strains are associated with a delay in the commencement of suitable antibiotic therapy, which consequently lengthens hospital stay and raises hospital costs [23]. Failure of antibiotic therapy is responsible for higher mortality rates in patients infected with these bacteria [24].

Epidemiological studies around the world have investigated the prevalence of ESBL-producing *Enterobacteriaceae* and they have seen multiple mechanisms of drug-resistance [25–33]. Several studies on ESBL infection in Asian-pacific region reported 60–80% of such cases were nosocomial-acquired while, remaining were community-acquired infections [1, 3, 34–39]. Over the last decade in Pakistan, an increase in resistance against quinolones has been observed in *Enterobacteriaceae* [40]. However, not much is known about fluoroquinolone-resistance in ESBLs and its relationship with plasmid-encoded genes.

MDRs are posing a treatment challenge, and are emerging as a major cause of morbidity and mortality worldwide. Unfortunately, proper surveillance and documentation of such pathogens is very limited, especially in developing countries. It has been estimated that more than 70% of antibiotic resistance occurs in the Asia-pacific region of the world, making antimicrobial resistance extremely problematic for Asian countries [1]. In Pakistan, ESBLs are especially problematic in terms of their contribution to the MDR bacteria problem. From this perspective, we analysed all of the available data regarding the prevalence of ESBL-producing isolates in different regions of Pakistan.

Antimicrobial resistance is on the rise. There are many factors associated with increasing antimicrobial resistance, one of which is ESBL production. The distribution of ESBLs differs in different communities, and every community must design their own protocol regarding the prevention and treatment of such infections [41]. Developed countries have annual surveillance systems to monitor the impact of antibiotic resistance as well as to determine the causative agents of antibiotic resistant infections. Such surveillance systems are often inadequate in developing countries. This meta-analysis will improve understanding of the distribution and epidemiology of ESBLs with different gene variants in Pakistan. This study also highlights the need to use molecular techniques to determine the different gene variants associated with ESBL-producing bacteria in Pakistan. To our knowledge, this is the first meta-analysis report from Pakistan, which would aid in updating the national treatment guidelines for ESBL infections. The purpose of this study was to determine the pooled prevalence of ESBL-producing *Enterobacteriaceae* with different gene variants in Pakistan.

## Methods

### Study design

This is a descriptive, meta-analysis study and is comprised of different studies reported from within Pakistan.

### Literature search and strategy

A Web-based search using the key words: ESBLs, Pakistan, ESBL genes and *Enterobacteriaceae*, were performed using the electronic databases PubMed, Scopus, PakMedi Net and Web of Science in September 2016. Articles published in the English language were included in the study. A comprehensive search was carried out for publications on the subject of ESBLs from Pakistan. Furthermore, the references cited within the articles were also carefully screened to look for additional relevant publications.

### Study selection procedures and criteria

Study selection was carried out by three authors in three steps independently (SH, SA and SR). As a first step, all of the titles and abstracts that were related to the study question were reviewed, and these were included in a group of eligible articles with irrelevant articles being excluded. All articles in the initially selected group were further screened in a second step by reviewing the full details of the articles. As a third step, selected articles were evaluated by other authors specifically for meta-analysis (RAK, NA, H H, SR), which was conducted using software STATA version 14.1 (College Station, Texas, USA) as previously described [42].

All studies were included based on the following criteria 1) studies that reported the prevalence of ESBLs in any province of Pakistan; 2) studies on bacterial strains isolated from human specimen; 3) all relevant national and international full text original research articles; 4) studies with confirmed ESBLs using phenotypic detection methods; 5) studies that used molecular techniques for ESBL gene variants.

**Fig. 1:**
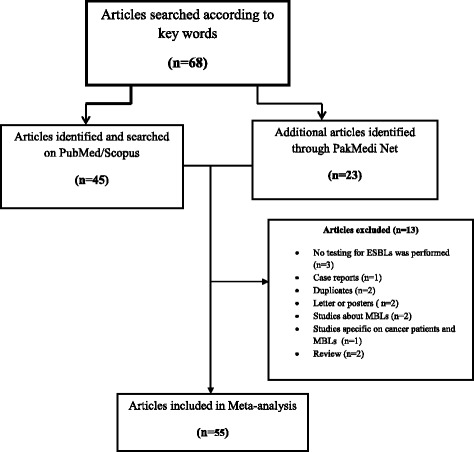
Flowchart of Systematic literature search and article selection

**Fig. 2:**
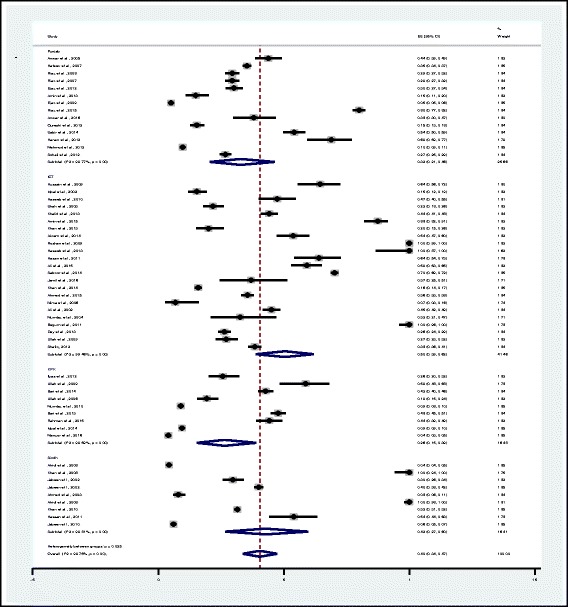
Proportion estimates of ESBL-producing *Enterobacteriaceae* in Pakistan. Midpoint of each horizontal line segment shows the proportion estimate of ESBL in each study. Rhombic mark shows the pooled proportion from all studies included

Studies were excluded based on the following criteria 1) studies with incomplete information related to phenotypic ESBL detection methods; 2) duplicate articles, case reports, very small datasets (few strains < 15), abstracts/titles only, posters and review articles 3) studies on β-lactamases other than ESBL; 4) studies on animals and environmental strains of non-human origin. After reading the full texts, 13 further articles were removed for miscellaneous reasons [no phenotypic testing for ESBLs was performed (*n* = 3), case reports (*n* = 1), duplicates (*n* = 2), letter or posters (*n* = 2), studies about MBLs (*n* = 2), Studies specific on cancer patients and (mettalo-β-lactamases) MBLs (*n* = 1) and reviews (*n* = 2)] (Figs. 1 and 2).

### Data extraction

The data were extracted by SH, SA and SR from the eligible studies and included. The data extracted from eligible studies consisted of; year of publication, year in which study was conducted, name of authors, location where the study was conducted (different provinces of Pakistan), sample size, strains detected ESBL, phenotypic detection techniques including; the double disc synergy test (DDST), the combination disc test (CDST), the epsilometric test (E-Test) and molecular detection techniques for gene variants (PCR) (Tables 1 and 2). Data were extracted and analysed twice to remove any discordance.

**Table 1:** Distribution of Articles reviewed regarding ESBL-producing clinical isolates in different regions of Pakistan

	Publication Year	Study Year	Authors	Sample size	ESBL positive	Phenotypic Detection Tests				Molecular detection tests	
						^a^DDST	^b^CDST	^c^E-Test	^d^MIC	Types of Genes detected	Most Prevalent
Punjab											
1	2002	2002	Shah et al., [76]	378	58	YES	NO	NO	NO	NONE	NA
2	2003	2002–2003	Shah et al., [77]	400	87	YES	NO	NO	NO	NONE	NA
3	2004	2002	Ali et al., [47]	812	366	YES	NO	NO	NO	NONE	NA
4	2006	2006	Mirza et al., [66]	59	4	YES	YES	YES	YES	NONE	NA
5	2007	2004–2006	Mumtaz et al., [78]	46	15	YES	NO	NO	NO	NONE	NA
6	2007	2005	Anwar et al., [12]	324	142	YES	No	NO	NO	NONE	NA
7	2009	2006	Amin et al., [15]	200	40	YES	NO	NO	YES	NONE	NA
8	2009	2007–2008	Hafeez et al., [6]	3099	1094	YES	No	NO	NO	NONE	NA
9	2009	2006–2007	Ullah et al., [20]	392	106	YES	NO	NO	NO	NONE	NA
10	2010	2002–2007	Khan et al., [79]	200	175	YES	NO	NO	NO	NONE	NA
11	2012	2007–2008	Riaz et al., [22]	1018	300	YES	YES	NO	NO	NONE	NA
12	2011	–	Riaz et al., [8]	1018	300	YES	YES	YES	NO	NONE	NA
13	2011	2009–2010	Ejaz et al., [11]	13,638	698	YES	No	NO	NO	NONE	NA
14	2011	2010	Hussain et al., [45]	121	78	YES	NO	NO	NO	AmpC, Class A β-lactamases	CTX-M
15	2011	2008–2009	Roshan et al., [80]	308	308	YES	YES	NO	NO	NONE	NA
16	2011	–	Hassan et al., [81]	100	64	YES	NO	NO	NO	NONE	NA
17	2012	2006–2009	Mehmod et al., [58]	4200	408	NO	NO	NO	NO	AmpCs	NA
18	2013	2005,2010	Habeeb et al., [82]	173	82	YES	YES	NO	NO	NONE	NA
19	2013	2011	Begum et al., [48]	91	91	YES	YES	NO	YES	NONE	NA
20	2013	2010–2012	Ejaz et al., [9]	710	214	YES	YES	NO	NO	NONE	NA
21	2013	2011–2012	Amin et al., [10]	221	33	YES	YES	NO	NO	NONE	NA
22	2013	2012	Qureshi et al., [13]	672	103	NO	YES	NO	NO	NONE	NA
23	2013	2009	Hanan et al., [44]	103	71	YES	NO	NO	NO	NONE	NA
24	2013	2009–2010	Khalid et al., [83]	824	364	YES	NO	NO	NO	NONE	NA
25	2013	2009–2010	Habeeb et al., [16]	25	25	YES	NO	NO	NO	TEM,SHV,OXA,CTX-M	TEM
26	2013	2011	Day et al., [69]	1140	300	YES	NO	NO	NO	CTX-M, SHV	CTXM
27	2013	2008	Shafiq et al., [18]	1328	511	NO	NO	NO	YES	NONE	NA
28	2014	–	Sabir et al., [84]	500	271	YES	NO	NO	NO	NONE	NA
29	2014	2011–2013	Kausar et al., [46]	225	121	YES	NO	NO	NO	NONE	NA
30	2014	2011–2013	Saboor et al., [85]	3851	2707	YES	NO	NO	NO	NONE	NA
31	2015	2012–2014	Sohail et al., [86]	1429	382	YES	NO	NO	NO	NONE	NA
32	2015	–	Khan et al., [14]	2400	381	NO	NO	YES	NO	NONE	NA
33	2015	–	Riaz et al., [7]	1018	815	YES	YES	YES	NO	TEM, SHV, OXA	OXA
34	2015	–	Ahmed et al., [87]	1362	484	YES	NO	NO	NO	NONE	NA
35	2016	2015–2016	Ali et al., [17]	250	148	YES	NO	NO	YES	*Qnr*genes	*qnrB*
36	2016	2014	Jamil et al., [88]	46	17	YES	NO	NO	NO	NONE	NA
37	2016	2005	Anwar et al., [89]	121	46	YES	YES	NO	NO	NONE	NA
KhyberPakhtunkhawa											
38	2009	2005–2006	Ullah et al., [20]	342	66	YES	YES	NO	NO	NONE	NA
39	2009	2006–2007	Ullah et al., [49]	92	54	YES	NO	NO	NO	NONE	NA
40	2011	2009	Mumtaz et al., [90]	4150	371	YES	NO	NO	NO	NONE	NA
41	2013	2013	Bari et al., [21]	1037	495	YES	NO	NO	NO	NONE	NA
42	2014	2012	Ilyas et al., [19]	195	50	YES	NO	NO	NO	NONE	NA
43	2014	2013	Bari et al., [21]	1037	443	YES	NO	NO	NO	NONE	NA
44	2014	–	Iqbal et al., [91]	4010	379	YES	NO	NO	NO	NONE	NA
45	2016	2013–2014	Rahman et al., [50]	355	157	YES	NO	NO	YES	TEM-1, CTX-M 1	CTX-M 1
46	2016	2010–2014	Ahmed et al., [59]	3450	138	YES	YES	NO	NO	NONE	NA
Sindh											
47	2003	2002	Jabeen et l., [92]	471	140	YES	YES	NO	NO	NONE	NA
48	2005	2002	Jabeen et l., [93]	2840	1137	YES	NO	NO	NO	NONE	NA
49	2009	–	Ahmed et al., [94]	500	40	YES	NO	NO	NO	NONE	NA
50	2010	2007–2008	Khan et al., [79]	65	65	YES	NO	YES	NO	NONE	NA
51	2010	1990–2006	Jabeen et al., [95]	1967	120	NO	NO	NO	YES	NONE	NA
52	2010	2002–2007	Khan et al., [96]	15,914	5016	NO	YES	NO	NO	NONE	NA
53	2011	2008	Afridi et al., [97]	4492	190	YES	NO	NO	NO	NONE	NA
54	2011	–	Hassan et al., [98]	100	54	NO	YES	NO	YES	NONE	NA
55	2012	2008	Afridi et al., [99]	190	190	YES	NO	NO	NO	NONE	NA

NA (Not applied)

^a^DDST (Double Disc Synergy Test)

^b^CDST (Combination Disc Test)

^c^E-Test (Epsilometric Test)

^d^MIC(Minimum Inhibitory Concentration)

**Table 2:** Proportion estimates of ESBLs in different regions of Pakistan

Study	ES [95% Conf. Interval]			% Weight
Punjab, Lahore				
Anwar et al., 2007 [12]	0.44	0.38	0.49	1.83
Hafeez et al., 2009 [6]	0.35	0.34	0.37	1.85
Amin et al., 2009 [15]	0.15	0.11	0.20	1.82
Riaz et al., 2011 [8]	0.29	0.27	0.32	1.84
Ejaz et al., 2011 [11]	0.05	0.05	0.06	1.85
Riaz et al., 2012 [22]	0.29	0.27	0.32	1.84
Mehmod et al., 2012 [58]	0.10	0.09	0.11	1.85
Ejaz et al., 2013 [9]	0.30	0.27	0.34	1.84
Qureshi et al., 2013 [13]	0.15	0.13	0.18	1.84
Hanan et al., 2013 [44]	0.69	0.59	0.78	1.79
Sabir et al., 2014 [84]	0.54	0.50	0.59	1.84
Sohail et al., 2015 [86]	0.27	0.24	0.29	1.84
Riaz et al., 2015 [7]	0.80	0.77	0.82	1.84
Anwar et al., 2016 [89]	0.38	0.29	0.47	1.8
Sub-total Random pooled ES	0.33	0.21	0.46	25.66
Punjab, Islamabad				
Shah et al., 2002 [76]	0.15	0.12	0.19	1.83
Shah et al., 2003 [77]	0.22	0.18	0.26	1.83
Ali et al., 2004 [47]	0.45	0.42	0.49	1.84
Mirza et al., 2006 [101]	0.07	0.02	0.16	1.74
Mumtaz et al., 2007 [78]	0.33	0.20	0.48	1.71
Ullah et al., 2009 [20]	0.27	0.23	0.32	1.83
Khan et al., 2010 [79]	0.20	0.15	0.26	1.82
Hussain et al., 2011 [45]	0.64	0.55	0.73	1.80
Roshan et al., 2011 [80]	1.00	0.99	1.00	1.83
Habeeb et al., 2013 [82]	0.47	0.40	0.55	1.81
Hassan et al., 2011 [81]	0.64	0.54	0.73	1.78
Begum et al., 2013 [48]	1.00	0.96	1.00	1.78
Day et al., 2013 [69]	0.26	0.24	0.29	1.84
Shafiq et al., 201 [18]	0.38	0.36	0.41	1.84
Amin et al., 2013 [10]	0.88	0.82	0.92	1.82
Khalid et al., 2013 [83]	0.44	0.41	0.48	1.84
Habeeb et al., 2013 [82]	1.00	0.86	1.00	1.62
Saboor et al., 2014 [84]	0.70	0.69	0.72	1.85
Akram et al., 2014 [5]	0.54	0.47	0.60	1.82
Khan et al., 2015 [14]	0.16	0.14	0.17	1.85
Ahmed et al., 2016 [59]	0.36	0.33	0.38	1.84
Ali et al., 2016 [47]	0.59	0.53	0.65	1.82
Jamil et al., 2016 [88]	0.37	0.23	0.52	1.71
Sub-total Random pooled ES	0.50	0.39	0.62	41.46
Khyber Pakhtunkhawa				
Ullah et al., 2009 [49]	0.19	0.15	0.24	1.83
Mumtaz et al., 2010 [90]	0.09	0.08	0.10	1.85
Ullah et al., 2010 [100]	0.59	0.48	0.69	1.78
Bari et al., 2013 [21]	0.48	0.45	0.51	1.84
Ilyas et al., 2014 [19]	0.26	0.20	0.32	1.82
Iqbal et al., 2014 [91]	0.09	0.09	0.10	1.85
Bari et al., 2014 [21]	0.43	0.40	0.46	1.84
Rahman et al., 2016 [50]	0.44	0.39	0.50	1.83
Ahmed et al., 2016 [59]	0.04	0.03	0.05	1.85
Sub-totaRandom pooled ES	0.26	0.15	0.39	16.48
Sindh				
Jabeen et l., 2003 [92]	0.30	0.26	0.34	1.83
Jabeen et l., 2005 [93]	0.40	0.38	0.42	1.85
Ahmed et al., 2009 [94]	0.08	0.06	0.11	1.84
Khan et al., 2010 [96]	1.00	0.94	1.00	1.75
Jabeen et l., 2010 [95]	0.06	0.05	0.07	1.85
Khan et al., 2010 [96]	0.32	0.31	0.32	1.85
Afridi et al., 2011 [97]	0.04	0.04	0.05	1.85
Hassan et al., 2011 [98]	0.54	0.44	0.64	1.78
Afridi et al., 2012 [99]	1.00	0.98	1.00	1.81
Sub-total Random pooled ES	0.43	0.27	0.60	16.4
Overall Random pooled ES	0.40	0.34	0.47	100.00

### Statistical analysis and reporting

Statistical analysis was performed using the *Metaprop* command in STATA version 14.1 (College Station, Texas, USA) to pool the published data regarding the predominance of ESBLs in different regions of Pakistan. Statistical heterogeneity was calculated using the I^2^ statistic (measure of inconsistency) at the significance level of 5%. Heterogeneity was used to study the variation in studies using the I^2^ statistic*.* The *p*-values (typically considered significant at 0.05) were used for converting meta-analysis results to defined/known tests of statistics. Random-effects model (REM) was used to estimate the pooled prevalence and 95% CI. A funnel plot and Begg tests were performed to evaluate the publication partiality using data graphically and statistically.

## Results

### Distribution of articles describing ESBLs in Pakistan

**Fig. 3:**
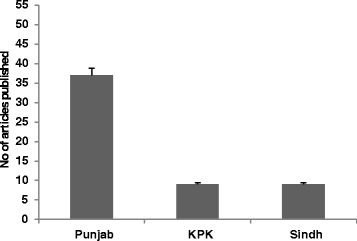
Distribution of articles in three regions of Pakistan

**Fig. 4:**
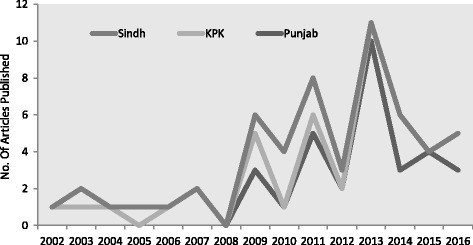
Annual publication of articles in different regions of Pakistan

Electronic database searches yielded a total of 68 studies. A total of 55 articles reviewed from four provinces of Pakistan included 14 (25.4%) from Punjab, 23 (41.8%) from the Islamabad/Rawalpindi, 9 (16.4%) from the KPK, while the remaining 9 (16.4%) were from the Sindh. No studies were found from Baluchistan province (Fig. 3 and Table 3). The maximum number of articles on this subject was published in year 2013, followed by 2011, with the number of published articles in Pakistan decreasing afterwards (Fig. 4). In total, 42 (76.4%) of the articles reviewed included cases from in-patient and out-patient departments (OPD), 10 (15%) included patients attending in-patient departments, and 3 (6.3%) included patients attending OPD. A total of 21, 232 ESBL-bacterial isolates were included in the analysis. A total of 53 (96.4%) of the reviewed studies were conducted on both adults and children, while only 2 (3.6%) studies were based solely on the paediatric population. No studies were found on male and females separately (Table 2).

**Table 3:** Distribution of published articles in different regions of Pakistan

Year	Punjab	KPK	Sindh	Annual Total Publications
2002	1 (2.7%)	0 (0%)	0 (0%)	1 (1.81%)
2003	1(2.7%)	0 (0%)	1 (11.1%)	2 (3.63%)
2004	1(2.7%)	0 (0%)	0(0%)	1 (1.81%)
2005	0(0%)	0 (0%)	1 (11.1%)	1 (1.81%)
2006	1(2.7%)	0 (0%)	0(0%)	1 (1.81%)
2007	2 (5.4%)	0 (0%)	0(0%)	2 (3.63%)
2008	0 (0%)	0 (0%)	0(0%)	0(0%)
2009	3 (8.1%)	2 (22.2%)	1 (11.1%)	6 (10.9%)
2010	1(2.7%)	0 (0%)	3 (33.3%)	4 (7.27%)
2011	5 (13.5%)	1 (11.1%)	2 (22.2%)	8 (14.5%)
2012	2 (5.4%)	0 (0%)	1 (11.1%)	3 (5.45%)
2013	10 (27%)	1 (11.1%)	0(0%)	11 (20%)
2014	3(8.1%)	3 (33.3%)	0(0%)	6 (10.9%)
2015	4(10.8%)	0 (0%)	0(0%)	4 (7.27%)
2016	3(8.1%)	2 (22.2%)	0(0%)	5 (9.09%)
Region-Wide	37	9	9	55

### Laboratory methods used to estimate the proportion of ESBLs

For the variable phenotype methods, out of 55 studies, 48 (87.3%) had performed the double disc synergism test (DDST). However, only 13 (23.6%) had performed the combination disc test (CDST), and only 5 (9.09%) had performed the epsilometric test (E-Test). None of the studies had used the broth minimum inhibitory concentration (MIC) method. Out of 55 studies, 28 (50.9%) were published in local journals while the remaining 27 (49.1%) were in international journals (Table 2).

### Molecular methods used to estimate the proportion of ESBLs

For molecular detection methods, 6 (11%) out of 55 articles reported PCR-based gene detection methods. Among these, 50% of articles reported CTX-M group as the most prevalent group, and CTXM-1 as the most commonly found gene variant. Additionally, CTX-M and TEM combinations were found in 33% of selected articles. One study reported the association of *qnrB* genes with the appearance of the ESBLs phenotype (Table 1).

### Prevalence of ESBLs and their distribution in different geographical areas of Pakistan

Based on the available data (Table 1), Pakistan’s overall pooled proportion of ESBL-production was 0.40 (95% CI: 0.34–0.47). The overall heterogeneity was significant (I^2^ = 99.75%, *p* < 0.001), and significant ES = 0 (Z = 18.41, *p* < 0.001). The pooled proportion of ESBL-production for the Punjab, the Islamabad/Rawalpindi region, the KPK and the Sindh regions was 0.33 (95% CI: 0.21–0.46), 0.50 (95% CI: 0.39–0.62), 0.26 (95% CI: 0.15–0.39) and 0.43 (95% CI: 0.27–0.60) respectively. Significant heterogeneity (I^2^) for four regions with *p* < 0.001 is 99.77, 99.46, 99.59 and 99.81% respectively. Significant ES = 0 at *p* < 0.001 for the Punjab, the Islamabad/Rawalpindi, the KPK and the Sindh regions is Z = 8.32, 12.65, 7.43 and 7.87 respectively (Fig. 2 and Table 2).

## Discussion

To address the issue of MDR bacteria, it is necessary to raise awareness about the magnitude of the problem by collecting data about antibiotic-resistance in various countries and regions [1, 29, 43]. The scarcity of studies available from Pakistan warrants attention for future research. Limited data regarding the overall predominance of ESBLs from Pakistan are available, but with no studies specifically from Baluchistan. This is the first meta-analysis about the extent of the ESBL problem in the Pakistani population. This is the first meta-analysis regarding the extent of the ESBL problem in the Pakistani population. However, this meta-analysis finds a high percentage of ESBL-producing *Enterobacteriaceae* across different geographical regions of Pakistan [5, 7, 12, 20, 44–50].

This meta-analysis is comprised of different studies reported from within Pakistan. The overall pooled proportion for ESBLs in this meta-analysis for Pakistan was 40% (Table 2). In China, a nationwide survey comprised of 30 different hospitals reported a 46% ESBL proportion, which is quite close to the data reported in our study [51]. A survey conducted in the hospitals of East Africa reported an overall pooled ESBL proportion of 42% (95% CI: 0.34–0.50) [52]. Previous research showed a considerably higher frequency of ESBL in Asian and African countries compared to developed countries [53]. For instance, the German population showed the estimated ESBL proportion in the range of 10 to 15% [54]. Similarly, a report was published from the US in 2012, which was based on the surveillance of ESBLs in nine census regions of the US, and they reported 4 to 12% resistance due to ESBLs [55]. However, among the Asian continent, an increase in ESBL mediated resistance was observed among the Japanese community, where the pooled ESBL proportion increased from 6.3% to 20% in 9 years [56].

These results indicate an extensive and statistically significant degree of disparity in ESBL proportion estimates (*p* < 0.05). The variation in ESBL occurrence reported in this systematic report may depend on several factors, including the socio-economic status of a society and the availability of antibiotics [57]. Moreover, differences in the sensitivity and specificity of the different methods applied in determining the proportions are also contributing factors. The majority of studies used purely phenotypic approaches, while some studies used molecular methods along with phenotypic testing [7, 9, 16, 17, 44, 47, 49, 58, 59]. Differences in ESBL proportions have been documented from all over the world established by hospital or community-based surveys [60–62]. In a study conducted in Ha’Emek Medical Center Israel> 50% ESBL prevalence was reported for community-acquired infections [63]. Whereas studies in Egypt, Cameroon, Bamako, Spain, China, Saudi Arabia, United Kingdom, United States, Latin America between 2004 to 2008 indicated a prevalence of ESBLs between 10 and 61% in different hospital and community settings [63–68].

Due to limited resources and a lack of infrastructure, only a few articles (11%) have investigated the molecular characterization and presence of ESBL encoding genes [4, 7, 16, 24, 29, 44, 45, 50, 51, 58]. OXA, SHV, TEM and CTX-M were the most commonly found gene variants in these studies for ESBLs [7, 16, 17, 50, 69]. The CTX-M group was found to be prevalent in 50% of studies reporting utilization of PCR-based molecular detection methods. The CTXM-15 gene variant of CTX-M group 1 has already been reported in many studies in the Asian continent [70–73]. In particular, CTXM and TEM is a common gene variant combination [7, 50]. One study reported the association of *qnrB* gene variants with the appearance of an ESBL phenotype, as this gene is involved in fluoroquinolone resistance [17]. However, few studies have reported the incidence of NDM genes, which are responsible for Carbapenem-resistance [52]. There are many reasons for variations in ESBL prevalence in the four studied regions of Pakistan. There may be substrate preferences, higher use of any specific class of antibiotics, co-resistances to other classes of antibiotics, poor health and diagnostic facilities [74, 75]. With this limited available information to hand, it is challenging to plan intensive and effective interventions for combating the problem of resistance.

## Conclusion and recommendations

This meta-analysis indicated that there is a high ESBL burden in Pakistan. Few papers are available that address the annual frequency of ESBLs and their distribution in different provinces of Pakistan. No paper is available regarding the frequency of ESBLs in Baluchistan. Only 6 papers that reported gene detection were found. Detection of gene variants in β-lactamase-producing bacteria is essential information for the appropriate and effective treatment of patients. This underscores an urgent demand for regular surveillance to address this antimicrobial resistance issue. National and regional guidelines would be based upon such surveillance in order to understand the annual ESBLs burden. Effective measures such as the establishment of active surveillance and infection control programmes, emphasizing hand hygiene together with coherent antibiotic policies in hospitals and clinics should be implemented to stop and manage the spread of ESBLs in hospitals and communities.

## Abbreviations

CDST: Combination disc test
DDST: Double disc synergy test
ESBL: Extended-spectrum β-lactamases
ESBLs: Extended-spectrum β-lactamase-producing strains
E-Test: epsilometric test
KPK: Khyber Pakhtunkhawa
MBLs: Mettalo-β-lactamases
MDR: Multidrug-resistant
MIC: Minimum inhibitory concentration
OPD: Out-patient department
